# The Role of Gut Microbiota in Atherosclerosis and Hypertension

**DOI:** 10.3389/fphar.2018.01082

**Published:** 2018-09-25

**Authors:** Junli Ma, Houkai Li

**Affiliations:** Functional Metabolomic and Gut Microbiome Laboratory, Institute of Interdisciplinary Integrative Medicine Research, Shanghai University of Traditional Chinese Medicine, Shanghai, China

**Keywords:** gut microbiota, cardiovascular disease, atherosclerosis, TMAO, bile acids, hypertension, SCFAs

## Abstract

In recent years, accumulating evidence has indicated the importance of gut microbiota in maintaining human health. Gut dysbiosis is associated with the pathogenesis of a number of metabolic diseases including obesity, type 2 diabetes mellitus (T2DM), non-alcoholic fatty liver disease (NAFLD), and cardiovascular diseases (CVDs). Indeed, CVD has become the leading cause of death worldwide, especially in developed countries. In this review, we mainly discuss the gut microbiota-involved mechanisms of CVD focusing on atherosclerosis and hypertension, two major risk factors for serious CVD. Then, we briefly discuss the prospects of gut microbiota-targeted therapeutic strategies for the treatment of CVD in the future.

## Introduction

Gut microbiota is the collection of bacteria that inhabit in the gastrointestinal tract producing a diverse ecosystem about 10^14^ microorganisms (Kamada et al., 2013). The majority of the gut microbiota is composed of five *phyla*, namely *Bacteroidetes, Firmicutes, Actinobacteria, Proteobacteria*, and *Cerrucomicrobia*, in which the relative abundance of *Bacteroidetes* and *Firmicutes phyla* is >90% (Qin et al., 2010). The homeostasis of gut microbiota is critical for maintaining human health (Hansen et al., 2015; Kamo et al., 2017; Tang et al., 2017), with gut dysbiosis contributing to the development of various diseases including cardiovascular disease (CVD; Wang et al., 2011; Emoto et al., 2017), obesity (Ley et al., 2006; Henao-Mejia et al., 2012), type 2 diabetes mellitus (T2DM; Cani et al., 2008; Khan et al., 2014; Pedersen et al., 2016), non-alcoholic fatty liver disease (NAFLD; Mouzaki et al., 2013; Zhu et al., 2013), and even some types of cancer (Gopalakrishnan et al., 2018; Tilg et al., 2018).

Cardiovascular disease is the leading cause of death worldwide, especially in developed countries, and encompasses multiple disorders including atherosclerosis, hypertension, stroke, and heart failure (Mozaffarian et al., 2016). Although genetic contributions are intimately involved, other factors such as nutrition and gut microbiota have also been implicated as the main risk factors for developing CVD. Wang et al. (2011) reported the gut microbiota-dependent mechanism of CVD, highlighting the intricate relationship between gut microbiota and CVD. Recently, gut dysbiosis has been recognized as an important factor contributing to the development of atherosclerosis and hypertension, two major risk factors for CVD (Lau et al., 2017). Consequently, gut microbiota-targeted therapy is a promising strategy to treat CVD (Koopen et al., 2016; Anbazhagan et al., 2017; Santisteban et al., 2017).

In this review, we extensively retrieved the publications on the topics of gut microbiota and CVD mainly published within the past 10 years through PubMed. We discuss the roles of gut microbiota implicated in the development of CVD, especially focusing on atherosclerosis and hypertension, and briefly summarize the recent advances of gut microbiota-targeted therapies for CVD.

## Gut Microbiota and Atherosclerosis

Atherosclerosis is the major risk factor for CVD, which is characterized by accumulation of cholesterol and recruitment of macrophages into artery walls, contributing to the formation of atherosclerotic plaques (Gui et al., 2012). Interestingly, recent studies have suggested that gut dysbiosis can also contribute to the development of atherosclerosis (Drosos et al., 2015; Gregory et al., 2015; Jie et al., 2017). Using shotgun sequencing of the gut metagenome in patients with or without symptomatic atherosclerosis, scientists found that the relative abundance of *Roseburia* and *Eubacterium* was lower, while *Collinsella* was higher in atherosclerosis patients compared to healthy controls (Karlsson et al., 2012). In addition, *Akkermansia muciniphila* was found to improve gut barrier functions and exert protective effects against atherosclerosis (Li et al., 2016). Although meta-analysis showed no significant benefit in coronary artery disease patients treated with antibiotics (Andraws et al., 2005), nevertheless, evidence is accumulating which indicates that gut microbiota play a causative role in atherosclerotic by modulating inflammation and the production of microbial metabolites (Kasahara et al., 2017).

### Gut Dysbiosis and Inflammation in Atherosclerosis

Inflammation is commonly involved in a number of diseases (Xu et al., 2003; Ding et al., 2010), including atherosclerosis, which is a classical chronic inflammatory disease (Gui et al., 2012). Gut epithelium is the first barrier of the host, which protects against the invasion of pathogens (Desai et al., 2016). Given its critical role in preventing the translocation of intestinal content, mainly bacterial components, the integrity of the gut barrier is essential for maintaining the health of the host. Intestinal permeability is associated with reduced expression of tight junction proteins, including zonula occludens-1 (ZO-1), claudin-1, and occludin, and an imbalance between intestinal epithelial cell death and regeneration (Wang H. et al., 2014; Chen W.Y. et al., 2017). If the intestinal epithelial barrier is impaired, the invasion of pathogen associated molecular patterns (PAMPs) drives an immune response and results in systemic and tissue-specific inflammation. Accordingly, impairments to the gut barrier integrity induced by gut dysbiosis have been suggested as risk factor for chronic inflammation in various diseases. It is noteworthy that lipopolysaccharide (LPS) and peptidoglycan are microbial components that are recognized as risk factors for CVD.

**FIGURE 1:**
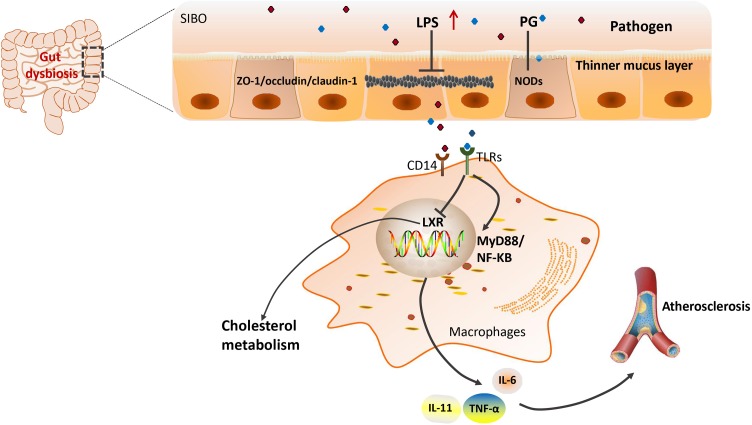
Gut microbiota and LPS-induced inflammation in atherosclerosis. LPS, lipopolysaccharides; ZO-1/occludin, two tight junction proteins; CD14, the monocyte differentiation antigen; TLR4, Toll-like receptor 4; LXR, liver X receptor; MyD88, myeloid differentiation primary response gene 88; NF-κB, nuclear factor kappa B; IL, interleukin; TNF-α, tumor necrosis factor-alpha; PG, peptidoglycan; NODs, nucleotide-binding oligomerization domain proteins.

Lipopolysaccharide is a cell wall component of Gram-negative [G(–)] bacteria, which has been extensively studied as it is one of the PAMPs involved in CVD risk. The association between LPS and CVD was first proposed in 1999 by measuring plasma endotoxin levels in the clinic (Wiedermann et al., 1999). Subsequently, the relationship was gradually confirmed by multiple experiments by different research groups (Niebauer et al., 1999; Stoll et al., 2004; Miller et al., 2009; Mitra et al., 2015). For example, in one study, it was concluded that the level of circulating endotoxemia was most notable in patients with the highest CVD burden (McIntyre et al., 2011). Cani et al. (2007) found that gut dysbiosis suppressed the expression of tight junction proteins, leading to an increase in intestinal permeability and subsequently the translocation of LPS into the blood (Harris et al., 2012). Gut dysbiosis-derived LPS may play important roles by modulation of Toll-like receptors (TLRs) and their downstream targets (Libby, 2002; Chacon et al., 2017). As part of the pattern-recognition receptors family, TLRs can recognize bacterial products and modulate the host immune system (Akira and Takeda, 2004; Akira et al., 2006). Circulating LPS can bind to cell-surface-receptor complexes composed of TLR4 and its co-receptors cluster of differentiation 14 (CD14; Neves et al., 2013). Using TLR4 and LDL receptors double knockout mice, Ding et al. (2012) found that a TLR4 deficiency reduced atherosclerosis without effect on inflammation (Ding et al., 2012). Consistently, clinical investigations have revealed that upregulation of TLRs was associated with inflammatory activation in human atherosclerosis, and promoted the development of atherosclerosis (Xu et al., 2001; Edfeldt et al., 2002). However, a meta-analysis in 2012 indicated that Asp299Gly, a TLR4 polymorphism, did not play an obvious role in the development of atherosclerosis (Zhang et al., 2012). Moreover, the binding of LPS to TLR4 activated its downstream pathways including MYD88 and nuclear factor kappa B (NF-κB), contributing to the increased production of pro-inflammatory cytokines such as IL-6, IL-1, IL-27, and tumor necrosis factor-alpha (TNF-α), leading to an increased risk of developing CVD (Barton and Kagan, 2009; Guzzo et al., 2012). Bjorkbacka et al. (2004) showed that a deficiency of MyD88 reduced atherosclerosis by decreasing macrophage recruitment (Bjorkbacka et al., 2004). The main interactions between gut microbiota and inflammation are shown schematically in **Figure 1**.

In addition, another bacterial PAMP, peptidoglycan (PG), was also found to be associated with CVD risk by impairing the intestinal epithelial barrier. PG is a minor cell wall component of [G(–)] bacteria; however, it is also a major component of Gram-positive [G(+)] bacteria. Using metagenomic sequencing, scientists found that patients with atherosclerosis had enrichment of genes that encoded PG synthesis (Karlsson et al., 2012). Indeed, pro-inflammatory bacterial PG was observed in atherosclerotic arteries and associated with vulnerable plaques (Laman et al., 2002). Through PG recognition, the nucleotide-binding oligomerization domain (NOD) proteins NOD1 and NOD2 promote intracellular bacteria clearance through a program involving NF-κB and mitogen-activated protein kinase (MAPK) signaling pathways (Philpott et al., 2014). Studies in Nod2-deficient mice revealed that NOD2 was a critical regulator of intestinal bacterial immunity and helps to maintain the integrity of the gut barrier (Kobayashi et al., 2005). In recent years, scientists have investigated the potential role of NOD1 in atherosclerosis using Nod1 knockout mice. Data showed that knockout of apolipoprotein E and Nod1 in mice significantly reduced the development of atherosclerotic lesions (Kanno et al., 2015). Additionally, there are other PAMPs that can promote inflammatory processes through the engagement of host pattern recognition receptors (PRRs), such as CpG oligodeoxynucleotides flagellin, lipopeptides, and so on (Kholy et al., 2015). Collectively, all of the evidence suggests that functional changes in the gut microbiota might be involved in the atherosclerosis risk. Although the vast majority of studies revealed that pathogenic bacteria contributed to atherosclerosis pathogenesis, two antibiotic trials reported controversial benefits of antibiotic therapy in CVD (Caligiuri et al., 2001; Munford, 2016).

### Gut Microbial Metabolites in Atherosclerosis

In addition to gut dysbiosis-related inflammation, increasing evidence has revealed that gut microbiota-derived metabolites play essential roles in the development of CVD (Brown and Hazen, 2015; Bergeron et al., 2016). A variety of metabolites are derived from the gut microbiota, as well as co-metabolism of gut microbiota such as amines methylamines, polyamines, short-chain fatty acids (SCFAs), trimethylamine N-oxide (TMAO), and secondary bile acids (BAs). SCFAs are a group of well-established gut microbial metabolites that are critically involved in metabolic diseases (Li et al., 2017). Recent advances detailing their involvement in atherosclerosis in both human and animal models have been extensively reviewed (Brown and Hazen, 2018). Therefore, in the current review, we mainly focused on the roles of TMAO and secondary BAs in atherosclerosis.

### TMAO and Atherosclerosis

Dietary phosphatidylcholine or L-carnitine is metabolized by gut microbiota into trimethylamine (TMA) in the intestine (Brown and Hazen, 2015). It is a precursor of TMAO, which is transported to liver and oxidized by flavin monooxygenase 3 (FMO3), one member of the hepatic FMO enzymes family, leading to the production of TMAO (Wang Z. et al., 2014). Hepatic knockdown of FMO3 in mice using an antisense oligonucleotide decreased circulating TMAO levels and attenuated atherosclerosis through stimulating basal metabolism and activating macrophage reverse cholesterol transport (RCT; Miao et al., 2015; Shih et al., 2015; Warrier et al., 2015). It was also found that plasma levels of gut microbial dietary phosphatidylcholine metabolites and TMAO that produced related molecules (L-carnitine and γ-butyrobetaine) were associated with the risk of CVD (Koeth et al., 2014; Chen K. et al., 2017; Guasch-Ferre et al., 2017). The higher level of plasma TMAO was correlated with atherosclerosis formation and the extent of the atherosclerotic plaque area (Wang et al., 2011). Consistently, a prospective and observational clinical study on patients with or without chronic heart failure has shown that plasma levels of TMAO were positively correlated with the risk of chronic heart failure (Troseid et al., 2015). These findings suggest that circulating levels of TMAO are important risk factors for the pathogenesis of CVD.

Given the roles of TMAO in the pathogenesis of CVD, the underlying mechanisms have been extensively investigated. To explore potential mechanisms by which TMAO might promote atherosclerosis, a dietary choline supplement was administered to ApoE^-/-^ mice, in which the expression of CD36 and steroid receptor RNA activator 1 (SR-A1), two macrophage scavenger receptors implicated in atherosclerosis, was measured. The results revealed elevated levels of CD36 and SR-A1 in the macrophages of TMAO-treated mice compared to normal controls, and antibiotic intervention reduced the formation of foam cells by decreasing TMA production (Wang et al., 2011). However, no significant impact of TMAO on foam cell formation was observed in mouse macrophages. In contrast, TMAO can lead to atherosclerosis by suppressing RCT and modulating the activity of cholesterol transporters in macrophages (Koeth et al., 2013). In addition, TMAO administration could suppress levels of liver BA synthetase (Cyp7a1 and Cyp27a1) and BA transporters (Oatp1, Oatp4, Mrp2, and Ntcp), leading to a disorder of BA-related pathways and atherosclerosis (Koeth et al., 2013), suggesting that the atherosclerotic promoting effect of TMAO is also associated with the variation in BA metabolism. Farnesoid X receptor (FXR) is an important nuclear receptor that controls BA metabolism, which can also regulate the expression of hepatic FMO3, resulting in an alteration in TMAO production (Bennett et al., 2013). An FXR agonist inhibited the expression of CYP7A1 and CYP8B1 in ApoE^-/-^ mice and protected mice against atherosclerosis (Mencarelli et al., 2009; Bennett et al., 2013; Miyazaki-Anzai et al., 2014; Miao et al., 2015). Recently, Ma et al. (2017) found that TMAO upregulated the expression of vascular cell adhesion molecule-1 (VCAM-1) and activated protein kinase C (PKC) and NF-κB, highlighting that TMAO may speed up the development of atherosclerosis by inducing endothelial cell dysfunction and by increasing monocyte adhesion. Additionally, the direct exposure of platelets to TMAO increased stimulus-dependent platelet activation by elevating Ca^2+^ release from intracellular stores, contributing to the increased risks of thrombosis and plaque instability (Zhu et al., 2016). Generally, TMAO accelerates the development of atherosclerosis by promoting cholesterol influx, inhibiting cholesterol efflux, blocking the BA pathway, and/or causing excessive activation of platelets. All of these findings confirmed TMAO as a biomarker for CVD risk and a promoter of atherosclerotic diseases (Senthong et al., 2016a,b; Zheng et al., 2016). TMAO is regarded as one of the most promising metabolites that may not only be an independent risk factor for CVD, but also a potential therapeutic target for CVD on the basis of a large amount of experimental and clinical data. However, inconsistent results were also observed, especially in large population observations (Dalmeijer et al., 2008; Nagata et al., 2015; Meyer et al., 2016). Choline is generally regarded as a dietary source of TMAO; however, in a cohort study, there was no clear evidence of significant associations between choline intake and the risk of developing CVD (Nagata et al., 2015). Likewise, in ApoE(-/-)mice, L-carnitine administration resulted in a significant increase in circulating TMAO levels, which surprisingly was inversely correlated with aortic lesion size(Collins et al., 2016). Unfortunately, several large population studies conducted by different countries have demonstrated that dietary choline and betaine intake was not associated with the pathogenesis of CVD (Bidulescu et al., 2007; Dalmeijer et al., 2008). Consequently, more studies are needed to confirm the exact roles of TMAO in atherosclerosis, as well as the validation of its therapeutic potential by targeting TMAO-producing bacteria or enzymes.

### Bile Acids and Atherosclerosis

Bile acids are another group of gut microbiota-derived metabolites involved in various metabolic diseases (Kuipers et al., 2014; Parseus et al., 2017), which are stored in the gallbladder and released into the intestine to facilitate the absorption of dietary lipids and fat-soluble vitamins. Primary BAs are synthesized from cholesterol in the liver and mainly include cholic acid (CA) and chenodeoxycholic acid (CDCA). Primary BAs are usually metabolized into secondary BAs including deoxycholic acid (DCA) and lithocholic acid (LCA), hyodeoxycholic acid, and ursodeoxycholic acid through gut microbiota-derived enzymes (Midtvedt, 1974; Russell, 2003). Previous studies reported that germ-free mice had higher levels of primary BAs, but non-detectable secondary BAs in the enterohepatic system (Sayin et al., 2013). It was found that suppression of hepatic BA biosynthesis could inhibit the HFD-induced gut microbiome alterations, which highlights the liver–BA–gut microbiome metabolic axis (Zheng et al., 2017). Thus, there is a bidirectional relationship between gut microbiota and BA metabolism (Jones et al., 2014).

Bile acids are also important signaling molecules that modulate host metabolism and energy expenditure processes (Dawson and Karpen, 2015; Joyce and Gahan, 2017). Bile salts can be diversified into biologically active species by gut microbiota that can survive in the bile salt-rich microenvironment. Gut microbiota-mediated BA metabolism in CVD has been well reviewed recently (Brown and Hazen, 2018). Nevertheless, to date, the role of BAs in CVD development is still poorly understood so far. It is well recognized that BAs can promote the development of atherosclerosis mainly through bile-salt hydrolase (BSH) and BA receptors (Lefebvre et al., 2009; Ridlon et al., 2016). The C24 N-acyl bond of glycine-conjugated or taurine-conjugated bile salts can be hydrolyzed into free BAs by BSH (Klaassen and Cui, 2015). In addition to deconjugation, the BA pool can also be chemically diversified by bacteria-derived 7α-dehydroxylase and 7β-dehydroxylase. The produced secondary BAs enter the portal circulation to function as signaling molecules with profound effects on host physiology and pathology (Lepercq et al., 2004). Bacteria-mediated BSH activity can affect the processes underlying the pathogenesis of atherosclerosis by increasing cholesterol accumulation, foam cell formation, and the size of the atherosclerotic plaque (Hansson et al., 2006). BSH is present in a wide range of bacteria such as *Methanobrevibacter smithii*, *Clostridium*, *Enterococcus*, and so on (Jones et al., 2012a; Tremaroli and Backhed, 2012).

In addition to BA itself, BA receptors are indispensable in mediating their biological functions. Farnesoid X-activated receptor (FXR) is one of the most important and well-studied BA receptors that regulates glucose and lipid metabolism by affecting transcription of genes that are involved in primary BA synthesis (Makishima et al., 1999; Wahlstrom et al., 2016). The critical role of FXR in mediating cholesterol metabolism was elucidated by using FXR^-/-^ mice which have increased plasma high density lipoprotein (HDL) cholesterol, non-HDL cholesterol and triglyceride levels compared to wild-type mice (Lambert et al., 2003). In a previous study, loss of functional FXR in apolipoprotein E-deficient (ApoE-/-) mice, a mouse model of atherosclerosis, resulted in more severe lipid metabolism defects and enhanced aortic plaque formation (Hanniman et al., 2005). Furthermore, FXR deficiency can result in a decrease of plasma low-density lipoprotein cholesterol and CD36 expression in macrophages, leading to a reduced risk of atherosclerosis in LDLR knockout (LDLR^-/-^) mice (Zhang et al., 2006). On the other hand, research indicates that activation of FXR with an agonist can protect against atherosclerosis in LDLR^-/-^ and ApoE^-/-^ mice, which may be associated with suppression of genes involved in BAs synthesis (Hartman et al., 2009). The G protein-coupled BA receptor, also known as TGR5, is another important host BA receptor that is responsive to BAs (Li and Chiang, 2015). Recent investigations have indicated that activation of TGR5 can inhibit atherosclerosis formation, an effect associated with a reduction of macrophage inflammation and lipid loading (Pols et al., 2011). Moreover, activation of TGR5 also contributes to enhanced energy expenditure and improved glycemic control (Watanabe et al., 2006). Pregnane X receptor (PXR) is another type of nuclear hormone receptor that regulates the expression of genes involved in the biosynthesis, transport, and metabolism of BAs, and can also be activated by secondary BAs such as LCA (Staudinger et al., 2001). Deletion of PXR attenuates the development of atherosclerosis in PXR and apoE double knockout (PXR^-/-^ and ApoE^-/-^) mice, which may be associated with the reduction of CD36 expression and lipid uptake in macrophages (Sui et al., 2011). It has been reported that activation of PXR by a PXR agonist increases the levels of atherogenic lipoproteins VLDL and LDL, and that PXR activation accelerates atherosclerosis in ApoE^-/-^ mice (Zhou et al., 2009). In addition, the vitamin D3 receptor (VDR) is a sensor for bacteria-induced BA that is much more sensitive to LCA and its metabolite (3-oxo-LCA) than other nuclear receptors (Makishima et al., 2002). It has been found that macrophage VDR signaling attenuates atherosclerosis in mice in part by inhibiting the local renin-angiotensin system (Szeto et al., 2012). Finally, sphingosine-1-phosphate receptor 2 (S1PR2) can be activated by various conjugated BAs and then promotes atherosclerosis by regulating macrophage retention and inflammatory cytokine secretion (Studer et al., 2012), whereas S1PR2 knockdown attenuates atherosclerosis in ApoE^-/-^ mice (Skoura et al., 2011).

**FIGURE 2:**
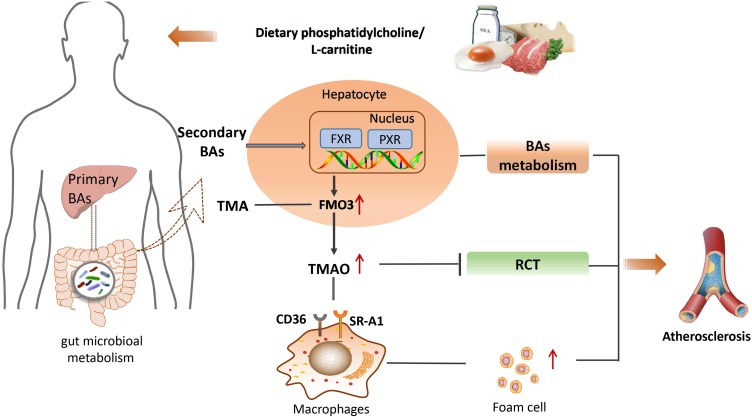
The main mechanisms between gut microbiota-derived metabolites and atherosclerosis. BA, bile acids; TMA, trimethylamine; FXR, farnesoid X receptor; PXR, pregnane X receptor; FMO3, flavin monooxygenase 3; TMAO, trimethylamine N-oxide; CD36, the monocyte differentiation antigen; RCT, reverse cholesterol transport; SR-A1, steroid receptor RNA activator 1.

In summary, gut microbiota-derived secondary BAs play important roles in the development of atherosclerosis through the modulation of various BA receptors such as FXR, PXR, TGR5, VDR, and S1PR2. This finding highlights the great potential for novel atherosclerosis therapy by targeting gut microbiota (Levi, 2016). The main mechanisms associated with gut microbiota-derived metabolites and atherosclerosis is shown schematically in **Figure 2**.

## Gut Microbiota and Hypertension

Hypertension is another important risk for CVD that is induced by both genetic susceptibility and environmental factors (Townsend et al., 2016). Given the increasing recognition of the role of gut microbiota in metabolic diseases (Karlsson et al., 2012; Tremaroli and Backhed, 2012; Jonsson and Backhed, 2017; Yamashiro et al., 2017), the relationship between gut microbiota and hypertension has also been evaluated in recent years. In 1982, it was demonstrated that antibiotic treatment could produce a higher blood pressure, which implicated the probable involvement of gut microbiota in regulating blood pressure (Honour, 1982). In spontaneously hypertensive rats, Yang et al. (2015) observed a significant decrease in microbial richness and diversity, and an increase in the ratio of *Firmicutes*/*Bacteroidetes*. In another study, compared with conventionally raised (CONV-R) mice, GF mice infused with AngII showed attenuation of the blood pressure increase in response to AngII, indicating that gut microbiota promotes AngII-induced vascular dysfunction and hypertension (Karbach et al., 2016). Accordingly, the gut microbiota is probably involved in the development of hypertension. Although the relationship and mechanism underlying gut microbiota and hypertension have not yet been fully elucidated, the existing evidence has highlighted the critical roles of SCFAs and oxidized low-density lipoprotein (ox-LDL) in hypertension.

### SCFAs and Hypertension

Short-chain fatty acids (such as acetate, proprionate, and butyrate), which are derived from dietary fiber (mainly polysaccharides), play crucial roles in maintaining the homeostasis of the gut microbiome and host immunity (El Kaoutari et al., 2013; Canfora et al., 2015; Koh et al., 2016; Miyamoto et al., 2016). Interestingly, bacteria that metabolize polysaccharides into different types of SCFAs are specific (Rey et al., 2010). For instance, the major acetate-producing bacteria are *Streptococcus* spp., *Prevotella* spp., *Bifidobacterium* spp., *Clostridiums* pp., *A. muciniphila*, and so on (Rey et al., 2010). Propionate is generated from carbohydrate fermentation by *Bacteroides* spp., *Salmonella* spp., *Dialister* spp., *Veillonella* spp., *Roseburia inulinivorans*, *Coprococcus catus*, *Blautia obeum*, etc. (Louis and Flint, 2017), while butyrate is derived from *Lachnospiraceae*, *Ruminococcaceae*, and *Acidaminococcaceae* families (Duncan et al., 2002). Clinical evidence has shown that the abundance of butyrate-producing bacteria is associated with a lower blood pressure in obese pregnant women (Gomez-Arango et al., 2016). A recent study found that fiber and acetate supplementation improved gut dysbiosis, associated with an increase in *Bacteroides acidifaciens*, which may play a protective role in hypertension and heart failure in hypertensive mice (Marques et al., 2017).

The role of host G-protein-coupled receptors (GPCRs) in the development of hypertension has been well reviewed (Pluznick et al., 2013). To date, there are at least three GPCRs that are regulated by SCFAs including GPR41, GPR43, and GPR109A (Tan et al., 2017). SCFAs can stimulate host GPCRs-regulated pathways to affect renin secretion and therefore blood pressure (Furusawa et al., 2013; Pluznick et al., 2013). One study has reported that GPR41 knockout mice exhibited systolic hypertension compared with wild-type mice, and that SCFAs lowered blood pressure by regulating endothelial GPR41 (Natarajan et al., 2016). Olfactory receptor 78 (Olfr78) is another type of GPCR expressed in the kidney, which can also be modulated by SCFAs such as acetate and propionate (Tan et al., 2017). In addition, both Olfr78 and GPR41 are expressed in smooth muscle cells of small resistance blood vessels (Pluznick et al., 2013). Propionate can induce vasodilation and produce an acute hypotensive response in mice through modulation of Olfr78 and GPR41 activity (Miyamoto et al., 2016). On the other hand, it was found that stimulation of GPR41 resulted in a reduction of the hypotensive response, and this effect could be opposed by stimulating Olfr78 (Pluznick, 2013). Interestingly, antibiotic treatment not only altered the composition of gut microbiota, but also increased blood pressure in Olfr78 knockout mice (Pluznick et al., 2013). In recent years, Reijnders et al. (2016) conducted a randomized double-blind placebo-controlled trial, in which SCFAs and a number of metabolic parameters were measured. The inconsistent outcome was reported that the levels of SCFAs had no significant effects on energy or glucose homeostasis in humans (Reijnders et al., 2016). Overall, although all these findings revealed that gut microbiota may play important roles in modulating the host blood pressure through production of microbial SCFAs, the potential for SCFAs to be a therapeutic target for CVD needs to be confirmed by additional investigations in the future.

### Oxidized Low Density Lipoprotein (ox-LDL) and Hypertension

Generally, the regulation of blood pressure depends on the magnitude of blood vessel vasoconstriction and vasodilation (Luscher and Barton, 1997). In addition to the regulation of various receptors, gut dysbiosis also contributes to hypertension through vasoconstriction mediated by oxidation of LDL (Packer et al., 2014).

Dysbiosis can promote the expression of pro-inflammatory cytokines and induce oxidative stress, which can stimulate Ox-LDL (Chawla et al., 2011; Peluso et al., 2012). Previous studies have shown that higher levels of oxLDL contribute to hypertension by inhibiting the production of nitric oxide (NO) and endothelin-1 (Subah Packer, 2007). NO is a well-established vasodilator that is produced through oxidation of L-arginine by NO synthase. Ox-LDL decreases the production of NO and reduces the degree of vasodilation (Ma et al., 2006). Moreover, endothelin-1 plays crucial roles in maintaining basic vascular tension and cardiovascular system homeostasis. Interestingly, the activity of endothelin-1 on blood vessels is concentration-dependent, that is, endothelin-1 produces vasodilatory effects at low concentrations by activating the endothelial receptor B (ETB) and promoting NO production, but produces vasoconstriction at high concentrations by increasing ox-LDL production in plaques and activating the endothelial receptor A (ETA; Boulanger and Luscher, 1990).

**FIGURE 3:**
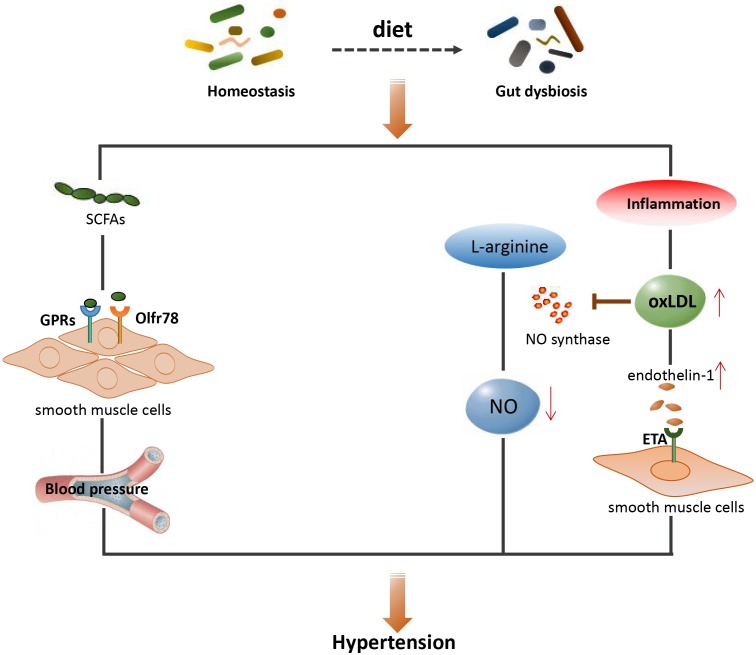
The main mechanisms between gut microbiota and hypertension. SCFAs, short-chain fatty acids; GPRs, G-protein-coupled receptors; Olfr78, olfactory receptor 78; NO, nitric oxide; OxLDL, oxidized low density lipoprotein; ETA, endothelin receptor A.

Although a causative relationship between gut dysbiosis and hypertension has been acquired (Kamo et al., 2017; Santisteban et al., 2017), the exact role of gut microbiota in mediating hypertension still requires further extensive investigation. The main mechanisms associated with gut microbiota and hypertension are shown schematically in **Figure 3**, together with a summary of microbial-derived metabolites and CVD development in **Table 1**.

**Table 1:** Gut microbial-derived metabolites and CVD.

Metabolite	Experimental models	Main observations	References
TMAO	FMO3 knockdown mice	The TMAO-generating enzyme FMO3 is a central regulator of cholesterol balance	Warrier et al., 2015
	Western diet (WD)-induced obese mice	Consumption of a WD increases circulating TMAO levels, which contributes to cardiac dysfunction	Chen K. et al., 2017
	C57BL/6 mice	TMAO promotes pathological process of atherosclerosis by impairing endothelial self-repair capacity and enhancing monocyte adhesion	Ma et al., 2017
	Apoe-/- female mice	Gut microbial metabolite γ-butyrobetaine is converted into TMA and TMAO, and accelerates atherosclerosis	Koeth et al., 2014
	Apoe-/- mice	Dietary choline or TMAO supplementation enhances atherosclerotic lesion development	Wang et al., 2011
	Apoe-/- mice	Dietary L-carnitine supplementation alters gut microbial composition, enhances production of TMA/TMAO, and increases atherosclerosis	Koeth et al., 2013
	Germ-free mice	Gut microbial metabolite TMAO enhances platelet hyperreactivity and thrombosis risk	Zhu et al., 2016
	ApoE(-/-) mice	L-carnitine intake and high plasma TMAO levels correlate with low aortic lesions	Collins et al., 2016
	155 patients with chronic heart failure	TMAO is associated with survival of patients with chronic heart failure	Troseid et al., 2015
	817 participants (young adults)	TMAO may not significantly contribute to early atherosclerotic disease risk	Meyer et al., 2016
	7447 participants (aged 55–80 years)	Plasma metabolites from choline pathway are associated with an increased risk of CVD	Guasch-Ferre et al., 2017
	4007 participants	Increased TMAO levels are associated with an increased risk of cardiovascular	Tang et al., 2013
	13,355 male and 15,724 female subjects	Choline and betaine intakes are not associated with CVD mortality risk	Nagata et al., 2015
	14,430 middle-aged subjects	No association exists between dietary choline intake and incident coronary heart disease	Bidulescu et al., 2007
	18 healthy participants	Gut microbe-generated TMAO from dietary choline is prothrombotic in subjects	Zhu et al., 2017
Bile acids	ApoE-/- and LDLR-/- mice	Dual activation of the bile acid nuclear receptor FXR and G-protein-coupled receptor TGR5 protects mice against atherosclerosis	Miyazaki-Anzai et al., 2014
	Germ-free (GF) mice	Gut microbiota inhibits bile acid synthesis in the liver by alleviating FXR inhibition	Sayin et al., 2013
	FXR-deficient (-/-) mouse	The function of FXR is associated with the potential to be pro-atherogenic	(Lambert et al., 2003)
	FXR-/- ApoE-/- mice	Loss of FXR function is associated with more extensive aortic plaque formation in atherosclerotic disease	Hanniman et al., 2005
	LDLR-/- mice	FXR deficiency causes reduced atherosclerosis	Zhang et al., 2006
	Fxr-/- Ldlr-/- (DKO) mice	Activation of FXR protects against atherosclerosis in mice	Hartman et al., 2009
	Ldlr(-/-)Tgr5(-/-) and Ldlr(-/-)Tgr5(+/+) mice	TGR5 activation inhibits atherosclerosis by reducing macrophage inflammation and lipid loading	Pols et al., 2011
	PXR(-/-) apoE(-/-) mice	Deficiency of PXR attenuates atherosclerosis development	Sui et al., 2011
	ApoE(-/-) mice	Activation of PXR accelerates atherosclerosis development	Zhou et al., 2009
	LDLR-/- VDR-/- mice	Macrophage VDR signaling inhibits atherosclerosis in part by suppressing the local renin-angiotensin system	Szeto et al., 2012
SCFAs	205 women	Blood pressure is associated with alterations in gut microbiota and production of butyrate	Gomez-Arango et al., 2016
	Hypertensive mice	Acetate supplementation changes the development of hypertension and heart failure	Marques et al., 2017
	Olfr78-/- mice	SCFAs produced by the gut microbiota modulate blood pressure via Olfr78 and Gpr41	Pluznick et al., 2013
	Gpr41 knockout mice	Microbial SCFAs lower blood pressure via endothelial GPR41	Natarajan et al., 2016

*SCFAs, short-chain fatty acids; TMAO, trimethylamine N-oxide; BAs, bile acids; Gpr41, G-protein-coupled receptor 41; Olfr78, olfactory receptor 78; FMO3, flavin monooxygenase 3; CD36, the monocyte differentiation antigen; FXR, farnesoid X receptor; ABCA1, ATP-binding membrane cassette transporter A1; LDL-R, lipoprotein receptor; TGR5, G-protein-coupled bile acid receptor; S1PR2, sphingosine-1-phosphate receptor-2; ApoE-/-, apolipoprotein E-deficient*.

## Gut Microbiota-Targeted Therapy of CVD

Given the contributions of gut microbiota to the development of CVD, they have emerged as a potentially important target for CVD therapy (Daliri et al., 2017; He and Shi, 2017). The most frequently used approaches to manipulate the gut microbiota include probiotic, prebiotic, natural components, fecal transplantation, and so on.

Probiotic is a collection of bacteria with a wide range of beneficial effects on host metabolism (Sanders, 2008; Ettinger et al., 2014; Yoo and Kim, 2016). The widely used probiotics are *Lactobacillus*, *Bifidobacterium*, and *Satreptococcus* (Kailasapathy and Chin, 2000; Miura and Ohnishi, 2014). In a randomized double-blind clinical trial, Fuentes et al. (2013) found that a probiotic of *Lactobacillus plantarum* CECT 7527, 7528, and 7529 reduced circulating cholesterol levels and inhibited the formation of atherosclerotic plaques in hyper-cholesterol patients. In another randomized control study, subjects taking *Lactobacillus reuteri NCIMB* 30242 showed more significant reductions of LDL-C and total cholesterol levels compared to subjects given placebo capsules (Jones et al., 2012b). In addition, the benefits of probiotics of different *Lactobacillus* bacteria (*Lactobacillus fermentum* CECT5716 (LC40), *Lactobacillus coryniformis* CECT5711 (K8) and *Lactobacillus gasseri* CECT5714 (LC9) in the regulation of blood pressure have been investigated in spontaneously hypertensive rats, and it was found that long-term administration of these probiotics could reduce systolic blood pressure (Gomez-Guzman et al., 2015). A recent study has reported that the probiotic *L. plantarum* ECGC13110402 was well tolerated and can be used as an alternative or supplement to reduce cardiovascular risk (Costabile et al., 2017).

Prebiotic is a class of indigestible food ingredients with benefits via selectively stimulating the growth of “good” and suppressing the growth of “bad” bacteria in the intestinal tract (Gibson and Roberfroid, 1995). Prebiotic can usually cause specific changes in the composition of gut microbiota and exert beneficial effects on host metabolism. Recent investigations have shown that an inulin-type fructans (ITFs) supplement improved endothelial function in ApoE^-/-^ mice, while administration of ITFs promoted the production of butyrate and resulted in atheroprotective effects(Watzl et al., 2005; Catry et al., 2018). A previous investigation reported that long-chain inulin could inhibit the formation of atherosclerotic plaque in ApoE^-/-^ mice, an effect that may be associated with alterations in lipid metabolism (Rault-Nania et al., 2006). In a randomized, single-blind, controlled crossover clinical trial, consumption of β-glucan altered the composition of gut microbiota, an effect associated with a reduction of CVD risk markers. Additionally, mannan oligosaccharide (MOS) is another type of prebiotic. In a recent study, a MOS supplement modulated the composition of gut microbiota, lowered plasma cholesterol levels, and improved atherosclerotic plaques in high cholesterol diet-fed mice (Hoving et al., 2018).

In addition to probiotic and prebiotic, some natural active ingredients from herbs also have protective or therapeutic actions on CVD by modulating the gut microbiota. For example, berberine is a well-studied herbal-derived chemical with effective activity against atherosclerosis. It was found that the anti-atherosclerotic effect of berberine was associated with the stimulation of *Akkermansia* in ApoE^-/-^ mice (Zhu et al., 2018). Another example is resveratrol that may have protective effect against several cardiovascular risk factors such as hyper-cholesterol and TMAO by modulating the gut microbiota and expression of genes involved in maintaining the integrity of the gut barrier (Bird et al., 2017). Moreover, resveratrol was found to attenuate TMAO-induced atherosclerosis by decreasing gut microbiota-mediated TMAO synthesis and increasing BA metabolism (Chen et al., 2016).

Fecal microbiota transplantation (FMT) is a promising method of introducing “healthy” bacteria from healthy subjects into the gastrointestinal tract of patients with dysfunctional guts, which has received much attention in recent years (Colman and Rubin, 2014). In one study, the insulin sensitivity of recipients was significantly enhanced after 6 weeks transfer of microbiota from lean normal donors to male recipients with metabolic syndrome. FMT increased the abundance of butyrate-producing bacteria suggesting that FMT is a potential strategy for CVD therapy (Vrieze et al., 2012). Nevertheless, the use of FMT is also limited in the clinic due to the possible risk of transferring endotoxins or infectious diseases to recipients (De Leon et al., 2013).

Although gut microbiota-targeted therapy to treat CVD is promising in the context of increasing positive experimental and clinical evidence, discrepant results have also been reported in both experimental and clinical studies. For instance, recently, scientists evaluated the effects of probiotic intervention on plasma TMAO levels in CKD patients, but there no significant change was observed after 3 months supplementation(Borges et al., 2018). Similarly, FMT from vegans resulted in a slight alteration in the composition of the gut microbiota, but no improvement in TMAO production or vascular inflammation (Smits et al., 2018).

## Conclusion

Although many types of medicines are available in the clinic to treat CVD, currently, it is still the leading cause of death worldwide. In recent years, increasing evidence has suggested an important role for gut microbiota in the development of both metabolic diseases and CVD. The findings have shed light on the great potential of targeting the gut microbiota to aid the elucidation of the fundamental mechanisms underlying disease and/or to uncover novel preventative or therapeutic regimes. Currently, most of the research efforts have focused on paid on establishing the relationship between gut dysbiosis and the development of CVD. Although much progress has been made, there is some way to go before the unequivocal establishment of gut microbiota-targeted therapy for CVD in the clinic.

Given the experimental and clinical advances with regard to the mechanisms of gut microbiota in the pathogenesis of CVD, there is great promise of finding new approaches to treat CVD by using gut microbial metabolites such as SCFAs and some types of BAs, or blocking the production of detrimental microbial metabolites such as TMAO with inhibitors. In addition, methods to alter the gut microbial composition with probiotic, prebiotic, natural components, and FMT should be further explored. In the future well-designed large-scale clinical studies will be needed to validate experimental and other small-scale preliminary clinical data. The integration of omics approaches (metabolomics, metagenomics, and metatranscriptomics) may be of critical significance to explore the exact roles of identified gut bacteria in the pathogenesis of many diseases.

## Author Contributions

JM drafted the manuscript. HL revised the manuscript.

## Conflict of Interest Statement

The authors declare that the research was conducted in the absence of any commercial or financial relationships that could be construed as a potential conflict of interest.

## References

[B1] Akira S. Takeda K. (2004). Toll-like receptor signalling. *Nat. Rev. Immunol.* 4 499–511. 10.1038/nri1391 15229469

[B2] Akira S. Uematsu S. Takeuchi O. (2006). Pathogen recognition and innate immunity. *Cell* 124 783–801. 10.1016/j.cell.2006.02.015 16497588

[B3] Anbazhagan A. N. Priyamvada S. Priyadarshini M. (2017). Gut microbiota in vascular disease: therapeutic target? *Curr. Vasc. Pharmacol.* 15 291–295. 10.2174/1570161115666170105095834 28056754

[B4] Andraws R. Berger J. S. Brown D. L. (2005). Effects of antibiotic therapy on outcomes of patients with coronary artery disease: a meta-analysis of randomized controlled trials. *JAMA* 293 2641–2647. 10.1001/jama.293.21.2641 15928286

[B5] Barton G. M. Kagan J. C. (2009). A cell biological view of Toll-like receptor function: regulation through compartmentalization. *Nat. Rev. Immunol.* 9 535–542. 10.1038/nri2587 19556980 PMC3934928

[B6] Bennett B. J. de Aguiar Vallim T. Q. Wang Z. Shih D. M. Meng Y. Gregory J. (2013). Trimethylamine-N-oxide, a metabolite associated with atherosclerosis, exhibits complex genetic and dietary regulation. *Cell Metab.* 17 49–60. 10.1016/j.cmet.2012.12.011 23312283 PMC3771112

[B7] Bergeron N. Williams P. T. Lamendella R. Faghihnia N. Grube A. Li X. (2016). Diets high in resistant starch increase plasma levels of trimethylamine-N-oxide, a gut microbiome metabolite associated with CVD risk. *Br. J. Nutr.* 116 2020–2029. 10.1017/s0007114516004165 27993177 PMC5885763

[B8] Bidulescu A. Chambless L. E. Siega-Riz A. M. Zeisel S. H. Heiss G. (2007). Usual choline and betaine dietary intake and incident coronary heart disease: the Atherosclerosis Risk in Communities (ARIC) study. *BMC Cardiovasc. Disord.* 7:20. 10.1186/1471-2261-7-20 17629908 PMC1934379

[B9] Bird J. K. Raederstorff D. Weber P. Steinert R. E. (2017). Cardiovascular and antiobesity effects of resveratrol mediated through the gut microbiota. *Adv. Nutr.* 8 839–849. 10.3945/an.117.016568 29141969 PMC5682996

[B10] Bjorkbacka H. Kunjathoor V. V. Moore K. J. Koehn S. Ordija C. M. Lee M. A. (2004). Reduced atherosclerosis in MyD88-null mice links elevated serum cholesterol levels to activation of innate immunity signaling pathways. *Nat. Med.* 10 416–421. 10.1038/nm1008 15034566

[B11] Borges N. A. Stenvinkel P. Bergman P. Qureshi A. R. Lindholm B. Moraes C. (2018). Effects of probiotic supplementation on trimethylamine-N-Oxide plasma levels in hemodialysis patients: a pilot study. *Probiotics Antimicrob Proteins.* [Epub ahead of print]. 10.1007/s12602-018-9411-1 29651635

[B12] Boulanger C. Luscher T. F. (1990). Release of endothelin from the porcine aorta, inhibition by endothelium-derived nitric oxide. *J. Clin. Invest.* 85 587–590. 10.1172/jci114477 2153712 PMC296463

[B13] Brown J. M. Hazen S. L. (2015). The gut microbial endocrine organ: bacterially derived signals driving cardiometabolic diseases. *Annu. Rev. Med.* 66 343–359. 10.1146/annurev-med-060513-093205 25587655 PMC4456003

[B14] Brown J. M. Hazen S. L. (2018). Microbial modulation of cardiovascular disease. *Nat. Rev. Microbiol.* 16 171–181. 10.1038/nrmicro.2017.149 29307889 PMC5885760

[B15] Caligiuri G. Rottenberg M. Nicoletti A. Wigzell H. Hansson G. K. (2001). Chlamydia pneumoniae infection does not induce or modify atherosclerosis in mice. *Circulation* 103 2834–2838. 10.1161/01.CIR.103.23.2834 11401941

[B16] Canfora E. E. Jocken J. W. Blaak E. E. (2015). Short-chain fatty acids in control of body weight and insulin sensitivity. *Nat. Rev. Endocrinol.* 11 577–591. 10.1038/nrendo.2015.128 26260141

[B17] Cani P. D. Amar J. Iglesias M. A. Poggi M. Knauf C. Bastelica D. (2007). Metabolic endotoxemia initiates obesity and insulin resistance. *Diabetes Metab. Res. Rev.* 56:1761. 10.2337/db06-1491 17456850

[B18] Cani P. D. Bibiloni R. Knauf C. Waget A. Neyrinck A. M. Delzenne N. M. (2008). Changes in gut microbiota control metabolic endotoxemia-induced inflammation in high-fat diet-induced obesity and diabetes in mice. *Diabetes Metab. Res. Rev.* 57 1470–1481. 10.2337/db07-1403 18305141

[B19] Catry E. Bindels L. B. Tailleux A. Lestavel S. Neyrinck A. M. Goossens J. F. (2018). Targeting the gut microbiota with inulin-type fructans: preclinical demonstration of a novel approach in the management of endothelial dysfunction. *Gut* 67 271–283. 10.1136/gutjnl-2016-313316 28377388 PMC5868295

[B20] Chacon M. R. Lozano-Bartolome J. Portero-Otin M. Rodriguez M. M. Xifra G. Puig J. (2017). The gut mycobiome composition is linked to carotid atherosclerosis. *Benef. Microbes* 9 1–14. 10.3920/bm2017.0029 29124969

[B21] Chawla A. Nguyen K. D. Goh Y. P. (2011). Macrophage-mediated inflammation in metabolic disease. *Nat. Rev. Immunol.* 11 738–749. 10.1038/nri3071 21984069 PMC3383854

[B22] Chen K. Zheng X. Feng M. Li D. Zhang H. (2017). Gut microbiota-dependent metabolite trimethylamine n-oxide contributes to cardiac dysfunction in western diet-induced obese mice. *Front. Physiol.* 8:139. 10.3389/fphys.2017.00139 28377725 PMC5359299

[B23] Chen W. Y. Wang M. Zhang J. Barve S. S. McClain C. J. Joshi-Barve S. (2017). Acrolein disrupts tight junction proteins and causes endoplasmic reticulum stress-mediated epithelial cell death leading to intestinal barrier dysfunction and permeability. *Am. J. Pathol.* 187 2686–2697. 10.1016/j.ajpath.2017.08.015 28935573 PMC5818631

[B24] Chen M. L. Yi L. Zhang Y. Zhou X. Ran L. Yang J. (2016). Resveratrol attenuates trimethylamine-N-Oxide (TMAO)-induced atherosclerosis by regulating TMAO synthesis and bile acid metabolism via remodeling of the gut microbiota. *MBio* 7:e02210-15. 10.1128/mBio.02210-15 27048804 PMC4817264

[B25] Collins H. L. Drazul-Schrader D. Sulpizio A. C. Koster P. D. Williamson Y. Adelman S. J. (2016). L-Carnitine intake and high trimethylamine N-oxide plasma levels correlate with low aortic lesions in ApoE(-/-) transgenic mice expressing CETP. *Atherosclerosis* 244 29–37. 10.1016/j.atherosclerosis.2015.10.108 26584136

[B26] Colman R. J. Rubin D. T. (2014). Fecal microbiota transplantation as therapy for inflammatory bowel disease: a systematic review and meta-analysis. *J. Crohns Colitis* 8 1569–1581. 10.1016/j.crohns.2014.08.006 25223604 PMC4296742

[B27] Costabile A. Buttarazzi I. Kolida S. Quercia S. Baldini J. Swann J. R. (2017). An in vivo assessment of the cholesterol-lowering efficacy of *Lactobacillus plantarum* ECGC 13110402 in normal to mildly hypercholesterolaemic adults. *PLoS One* 12:e0187964. 10.1371/journal.pone.0187964 29228000 PMC5724841

[B28] Daliri E. B. Lee B. H. Oh D. H. (2017). Current perspectives on antihypertensive probiotics. *Probiot. Antimicrob. Proteins* 9 91–101. 10.1007/s12602-016-9241-y 27900619

[B29] Dalmeijer G. W. Olthof M. R. Verhoef P. Bots M. L. van der Schouw Y. T. (2008). Prospective study on dietary intakes of folate, betaine, and choline and cardiovascular disease risk in women. *Eur. J. Clin. Nutr.* 62 386–394. 10.1038/sj.ejcn.1602725 17375117

[B30] Dawson P. A. Karpen S. J. (2015). Intestinal transport and metabolism of bile acids. *J. Lipid Res.* 56 1085–1099. 10.1194/jlr.R054114 25210150 PMC4442867

[B31] De Leon L. M. Watson J. B. Kelly C. R. (2013). Transient flare of ulcerative colitis after fecal microbiota transplantation for recurrent *Clostridium difficile* infection. *Clin. Gastroenterol. Hepatol.* 11 1036–1038. 10.1016/j.cgh.2013.04.045 23669309

[B32] Desai M. S. Seekatz A. M. Koropatkin N. M. Kamada N. Hickey C. A. Wolter M. (2016). A dietary fiber-deprived gut microbiota degrades the colonic mucus barrier and enhances pathogen susceptibility. *Cell* 167 1339.e1321–1353.e1321. 10.1016/j.cell.2016.10.043 27863247 PMC5131798

[B33] Ding S. Chi M. M. Scull B. P. Rigby R. Schwerbrock N. M. J. Magness S. (2010). High-fat diet: bacteria interactions promote intestinal inflammation which precedes and correlates with obesity and insulin resistance in mouse. *PLoS One* 5:e12191. 10.1371/journal.pone.0012191 20808947 PMC2922379

[B34] Ding Y. Subramanian S. Montes V. N. Goodspeed L. Wang S. Han C. (2012). Toll-like receptor 4 deficiency decreases atherosclerosis but does not protect against inflammation in obese low-density lipoprotein receptor-deficient mice. *Arterioscler. Thromb. Vasc. Biol.* 32 1596–1604. 10.1161/atvbaha.112.249847 22580897 PMC3748807

[B35] Drosos I. Tavridou A. Kolios G. (2015). New aspects on the metabolic role of intestinal microbiota in the development of atherosclerosis. *Metabolism* 64 476–481. 10.1016/j.metabol.2015.01.007 25676802

[B36] Duncan S. H. Barcenilla A. Stewart C. S. Pryde S. E. Flint H. J. (2002). Acetate utilization and butyryl coenzyme A (CoA):acetate-CoA transferase in butyrate-producing bacteria from the human large intestine. *Appl. Environ. Microbiol.* 68 5186–5190. 10.1128/AEM.68.10.5186-5190.2002 12324374 PMC126392

[B37] Edfeldt K. Swedenborg J. Hansson G. K. Yan Z. Q. (2002). Expression of toll-like receptors in human atherosclerotic lesions: a possible pathway for plaque activation. *Circulation* 105 1158–1161. 11889007

[B38] El Kaoutari A. Armougom F. Gordon J. I. Raoult D. Henrissat B. (2013). The abundance and variety of carbohydrate-active enzymes in the human gut microbiota. *Nat. Rev. Microbiol.* 11 497–504. 10.1038/nrmicro3050 23748339

[B39] Emoto T. Yamashita T. Kobayashi T. Sasaki N. Hirota Y. Hayashi T. (2017). Characterization of gut microbiota profiles in coronary artery disease patients using data mining analysis of terminal restriction fragment length polymorphism: gut microbiota could be a diagnostic marker of coronary artery disease. *Heart Vessels* 32 39–46. 10.1007/s00380-016-0841-y 27125213

[B40] Ettinger G. MacDonald K. Reid G. Burton J. P. (2014). The influence of the human microbiome and probiotics on cardiovascular health. *Gut Microbes* 5 719–728. 10.4161/19490976.2014.983775 25529048 PMC4615746

[B41] Fuentes M. C. Lajo T. Carrion J. M. Cune J. (2013). Cholesterol-lowering efficacy of *Lactobacillus plantarum* CECT 7527, 7528 and 7529 in hypercholesterolaemic adults. *Br. J. Nutr.* 109 1866–1872. 10.1017/s000711451200373x 23017585

[B42] Furusawa Y. Obata Y. Fukuda S. Endo T. A. Nakato G. Takahashi D. (2013). Commensal microbe-derived butyrate induces the differentiation of colonic regulatory T cells. *Nature* 504 446–450. 10.1038/nature12721 24226770

[B43] Gibson G. R. Roberfroid M. B. (1995). Dietary modulation of the human colonic microbiota: introducing the concept of prebiotics. *J. Nutr.* 125 1401–1412. 7782892 10.1093/jn/125.6.1401

[B44] Gomez-Arango L. F. Barrett H. L. McIntyre H. D. Callaway L. K. Morrison M. Dekker Nitert M. (2016). Increased systolic and diastolic blood pressure is associated with altered gut microbiota composition and butyrate production in early pregnancy. *Hypertension* 68 974–981. 10.1161/hypertensionaha.116.07910 27528065

[B45] Gomez-Guzman M. Toral M. Romero M. Jimenez R. Galindo P. Sanchez M. (2015). Antihypertensive effects of probiotics *Lactobacillus* strains in spontaneously hypertensive rats. *Mol. Nutr. Food Res.* 59 2326–2336. 10.1002/mnfr.201500290 26255877

[B46] Gopalakrishnan V. Helmink B. A. Spencer C. N. Reuben A. Wargo J. A. (2018). The influence of the gut microbiome on cancer, immunity, and cancer immunotherapy. *Cancer Cell* 33 570–580. 10.1016/j.ccell.2018.03.015 29634945 PMC6529202

[B47] Gregory J. C. Buffa J. A. Org E. Wang Z. Levison B. S. Zhu W. (2015). Transmission of atherosclerosis susceptibility with gut microbial transplantation. *J. Biol. Chem.* 290 5647–5660. 10.1074/jbc.M114.618249 25550161 PMC4342477

[B48] Guasch-Ferre M. Hu F. B. Ruiz-Canela M. Bullo M. Toledo E. Wang D. D. (2017). Plasma metabolites from choline pathway and risk of cardiovascular disease in the PREDIMED (Prevention With Mediterranean Diet) Study. *J. Am. Heart Assoc.* 6:e006524. 10.1161/jaha.117.006524 29080862 PMC5721752

[B49] Gui T. Shimokado A. Sun Y. Akasaka T. Muragaki Y. (2012). Diverse roles of macrophages in atherosclerosis: from inflammatory biology to biomarker discovery. *Mediat. Inflamm.* 2012:693083. 10.1155/2012/693083 22577254 PMC3337637

[B50] Guzzo C. Ayer A. Basta S. Banfield B. W. Gee K. (2012). IL-27 enhances LPS-induced proinflammatory cytokine production via upregulation of TLR4 expression and signaling in human monocytes. *J. Immunol.* 188 864–873. 10.4049/jimmunol.1101912 22156348

[B51] Hanniman E. A. Lambert G. McCarthy T. C. Sinal C. J. (2005). Loss of functional farnesoid X receptor increases atherosclerotic lesions in apolipoprotein E-deficient mice. *J. Lipid Res.* 46 2595–2604. 10.1194/jlr.M500390-JLR200 16186601

[B52] Hansen T. H. Gobel R. J. Hansen T. Pedersen O. (2015). The gut microbiome in cardio-metabolic health. *Genome Med.* 7:33. 10.1186/s13073-015-0157-z 25825594 PMC4378584

[B53] Hansson G. K. Robertson A. K. Soderberg-Naucler C. (2006). Inflammation and atherosclerosis. *Annu. Rev. Pathol.* 1 297–329. 10.1146/annurev.pathol.1.110304.100100 18039117

[B54] Harris K. Kassis A. Major G. Chou C. J. (2012). Is the gut microbiota a new factor contributing to obesity and its metabolic disorders? *J. Obes.* 2012:879151. 10.1155/2012/879151 22315672 PMC3270440

[B55] Hartman H. B. Gardell S. J. Petucci C. J. Wang S. Krueger J. A. Evans M. J. (2009). Activation of farnesoid X receptor prevents atherosclerotic lesion formation in LDLR-/- and apoE-/- mice. *J. Lipid Res.* 50 1090–1100. 10.1194/jlr.M800619-JLR200 19174369 PMC2681391

[B56] He M. Shi B. (2017). Gut microbiota as a potential target of metabolic syndrome: the role of probiotics and prebiotics. *Cell Biosci.* 7:54. 10.1186/s13578-017-0183-1 29090088 PMC5655955

[B57] Henao-Mejia J. Elinav E. Jin C. Hao L. Mehal W. Z. Strowig T. (2012). Inflammasome-mediated dysbiosis regulates progression of NAFLD and obesity. *Nature* 482 179–185. 10.1038/nature10809 22297845 PMC3276682

[B58] Honour J. (1982). The possible involvement of intestinal bacteria in steroidal hypertension. *Endocrinology* 110 285–287. 10.1210/endo-110-1-285 7053989

[B59] Hoving L. R. Katiraei S. Heijink M. Pronk A. van der Wee-Pals L. Streefland T. (2018). Dietary mannan oligosaccharides modulate gut microbiota, increase fecal bile acid excretion, and decrease plasma cholesterol and atherosclerosis development. *Mol. Nutr. Food Res.* 62:e1700942. 10.1002/mnfr.201700942 29665623 PMC6001637

[B60] Jie Z. Xia H. Zhong S. L. Feng Q. Li S. Liang S. (2017). The gut microbiome in atherosclerotic cardiovascular disease. *Nat. Commun.* 8:845. 10.1038/s41467-017-00900-1 29018189 PMC5635030

[B61] Jones M. L. Martoni C. J. Ganopolsky J. G. Labbe A. Prakash S. (2014). The human microbiome and bile acid metabolism: dysbiosis, dysmetabolism, disease and intervention. *Exp. Opin. Biol. Ther.* 14 467–482. 10.1517/14712598.2014.880420 24479734

[B62] Jones M. L. Martoni C. J. Parent M. Prakash S. (2012a). Cholesterol-lowering efficacy of a microencapsulated bile salt hydrolase-active *Lactobacillus reuteri* NCIMB 30242 yoghurt formulation in hypercholesterolaemic adults. *Br. J. Nutr.* 107 1505–1513. 10.1017/s0007114511004703 22067612

[B63] Jones M. L. Martoni C. J. Prakash S. (2012b). Cholesterol lowering and inhibition of sterol absorption by *Lactobacillus reuteri* NCIMB 30242: a randomized controlled trial. *Eur. J. Clin. Nutr.* 66 1234–1241. 10.1038/ejcn.2012.126 22990854

[B64] Jonsson A. L. Backhed F. (2017). Role of gut microbiota in atherosclerosis. *Nat. Rev. Cardiol.* 14 79–87. 10.1038/nrcardio.2016.183 27905479

[B65] Joyce S. A. Gahan C. G. (2017). Disease-associated changes in bile acid profiles and links to altered gut microbiota. *Dig. Dis.* 35 169–177. 10.1159/000450907 28249284

[B66] Kailasapathy K. Chin J. (2000). Survival and therapeutic potential of probiotic organisms with reference to *Lactobacillus acidophilus* and *Bifidobacterium* spp. *Immunol. Cell Biol.* 78 80–88. 10.1046/j.1440-1711.2000.00886.x 10651933

[B67] Kamada N. Seo S. U. Chen G. Y. Nunez G. (2013). Role of the gut microbiota in immunity and inflammatory disease. *Nat. Rev. Immunol.* 13 321–335. 10.1038/nri3430 23618829

[B68] Kamo T. Akazawa H. Suda W. Saga-Kamo A. Shimizu Y. Yagi H. (2017). Dysbiosis and compositional alterations with aging in the gut microbiota of patients with heart failure. *PLoS One* 12:e0174099. 10.1371/journal.pone.0174099 28328981 PMC5362204

[B69] Kanno S. Nishio H. Tanaka T. Motomura Y. Murata K. Ihara K. (2015). Activation of an innate immune receptor, Nod1, accelerates atherogenesis in Apoe-/- mice. *J. Immunol.* 194 773–780. 10.4049/jimmunol.1302841 25488987

[B70] Karbach S. H. Schonfelder T. Brandao I. Wilms E. Hormann N. Jackel S. (2016). Gut microbiota promote angiotensin ii-induced arterial hypertension and vascular dysfunction. *J. Am. Heart Assoc.* 5:e003698. 10.1161/jaha.116.003698 27577581 PMC5079031

[B71] Karlsson F. H. Fak F. Nookaew I. Tremaroli V. Fagerberg B. Petranovic D. (2012). Symptomatic atherosclerosis is associated with an altered gut metagenome. *Nat. Commun.* 3:1245. 10.1038/ncomms2266 23212374 PMC3538954

[B72] Kasahara K. Tanoue T. Yamashita T. Yodoi K. Matsumoto T. Emoto T. (2017). Commensal bacteria at the crossroad between cholesterol homeostasis and chronic inflammation in atherosclerosis. *J. Lipid Res.* 58 519–528. 10.1194/jlr.M072165 28130274 PMC5335582

[B73] Khan M. T. Nieuwdorp M. Backhed F. (2014). Microbial modulation of insulin sensitivity. *Cell Metab.* 20 753–760. 10.1016/j.cmet.2014.07.006 25176147

[B74] Kholy K. E. Genco R. J. Van Dyke T. E. (2015). Oral infections and cardiovascular disease. *Trends Endocrinol. Metab.* 26 315–321. 10.1016/j.tem.2015.03.001 25892452

[B75] Klaassen C. D. Cui J. Y. (2015). Review: mechanisms of how the intestinal microbiota alters the effects of drugs and bile acids. *Drug Metab. Dispos.* 43 1505–1521. 10.1124/dmd.115.065698 26261286 PMC4576672

[B76] Kobayashi K. S. Chamaillard M. Ogura Y. Henegariu O. Inohara N. Nunez G. (2005). Nod2-dependent regulation of innate and adaptive immunity in the intestinal tract. *Science* 307 731–734. 10.1126/science.1104911 15692051

[B77] Koeth R. A. Levison B. S. Culley M. K. Buffa J. A. Wang Z. Gregory J. C. (2014). gamma-butyrobetaine is a proatherogenic intermediate in gut microbial metabolism of L-carnitine to TMAO. *Cell Metab.* 20 799–812. 10.1016/j.cmet.2014.10.006 25440057 PMC4255476

[B78] Koeth R. A. Wang Z. Levison B. S. Buffa J. A. Org E. Sheehy B. T. (2013). Intestinal microbiota metabolism of L-carnitine, a nutrient in red meat, promotes atherosclerosis. *Nat. Med.* 19 576–585. 10.1038/nm.3145 23563705 PMC3650111

[B79] Koh A. De Vadder F. Kovatcheva-Datchary P. Backhed F. (2016). From dietary fiber to host physiology: short-chain fatty acids as key bacterial metabolites. *Cell* 165 1332–1345. 10.1016/j.cell.2016.05.041 27259147

[B80] Koopen A. M. Groen A. K. Nieuwdorp M. (2016). Human microbiome as therapeutic intervention target to reduce cardiovascular disease risk. *Curr. Opin. Lipidol.* 27 615–622. 10.1097/mol.0000000000000357 27676197

[B81] Kuipers F. Bloks V. W. Groen A. K. (2014). Beyond intestinal soap–bile acids in metabolic control. *Nat. Rev. Endocrinol.* 10 488–498. 10.1038/nrendo.2014.60 24821328

[B82] Laman J. D. Schoneveld A. H. Moll F. L. van Meurs M. Pasterkamp G. (2002). Significance of peptidoglycan, a proinflammatory bacterial antigen in atherosclerotic arteries and its association with vulnerable plaques. *Am. J. Cardiol.* 90 119–123. 10.1016/S0002-9149(02)02432-3 12106839

[B83] Lambert G. Amar M. J. Guo G. Brewer H. B. Jr. Gonzalez F. J. (2003). The farnesoid X-receptor is an essential regulator of cholesterol homeostasis. *J. Biol. Chem.* 278 2563–2570. 10.1074/jbc.M209525200 12421815

[B84] Lau K. Srivatsav V. Rizwan A. Nashed A. Liu R. Shen R. (2017). Bridging the gap between gut microbial dysbiosis and cardiovascular diseases. *Nutrients* 9:E859. 10.3390/nu9080859 28796176 PMC5579652

[B85] Lefebvre P. Cariou B. Lien F. Kuipers F. Staels B. (2009). Role of bile acids and bile acid receptors in metabolic regulation. *Physiol. Rev.* 89 147–191. 10.1152/physrev.00010.2008 19126757

[B86] Lepercq P. Gerard P. Beguet F. Raibaud P. Grill J. P. Relano P. (2004). Epimerization of chenodeoxycholic acid to ursodeoxycholic acid by *Clostridium baratii* isolated from human feces. *FEMS Microbiol. Lett.* 235 65–72. 10.1016/j.femsle.2004.04.011 15158263

[B87] Levi M. (2016). Role of bile acid-regulated nuclear receptor FXR and G protein-coupled receptor TGR5 in regulation of cardiorenal syndrome (cardiovascular disease and chronic kidney disease). *Hypertension* 67 1080–1084. 10.1161/hypertensionaha.115.06417 27045028 PMC5291829

[B88] Ley R. E. Turnbaugh P. J. Klein S. Gordon J. I. (2006). Microbial ecology: human gut microbes associated with obesity. *Nature* 444 1022–1023. 10.1038/4441022a 17183309

[B89] Li J. Lin S. Vanhoutte P. M. Woo C. W. Xu A. (2016). Akkermansia muciniphila protects against atherosclerosis by preventing metabolic endotoxemia-induced inflammation in apoe-/- mice. *Circulation* 133 2434–2446. 10.1161/circulationaha.115.019645 27143680

[B90] Li T. Chiang J. Y. (2015). Bile acids as metabolic regulators. *Curr. Opin. Gastroenterol.* 31 159–165. 10.1097/mog.0000000000000156 25584736 PMC4332523

[B91] Li X. Shimizu Y. Kimura I. (2017). Gut microbial metabolite short-chain fatty acids and obesity. *Biosci. Microbiota Food Health* 36 135–140. 10.12938/bmfh.17-010 29038768 PMC5633527

[B92] Libby P. (2002). Inflammation in atherosclerosis. *Nature* 420 868–874. 10.1038/nature01323 12490960

[B93] Louis P. Flint H. J. (2017). Formation of propionate and butyrate by the human colonic microbiota. *Environ. Microbiol.* 19 29–41. 10.1111/1462-2920.13589 27928878

[B94] Luscher T. F. Barton M. (1997). Biology of the endothelium. *Clin. Cardiol.* 20(11 Suppl. 2), II-3–10. 10.1002/j.1932-8737.1997.tb00006.x PMC7509692 9422846

[B95] Ma F. X. Zhou B. Chen Z. Ren Q. Lu S. H. Sawamura T. (2006). Oxidized low density lipoprotein impairs endothelial progenitor cells by regulation of endothelial nitric oxide synthase. *J. Lipid Res.* 47 1227–1237. 10.1194/jlr.M500507-JLR200 16522925

[B96] Ma G. Pan B. Chen Y. Guo C. Zhao M. Zheng L. (2017). Trimethylamine N-oxide in atherogenesis: impairing endothelial self-repair capacity and enhancing monocyte adhesion. *Biosci. Rep.* 37:BSR20160244. 10.1042/bsr20160244 28153917 PMC5333780

[B97] Makishima M. Lu T. T. Xie W. Whitfield G. K. Domoto H. Evans R. M. (2002). Vitamin D receptor as an intestinal bile acid sensor. *Science* 296 1313–1316. 10.1126/science.1070477 12016314

[B98] Makishima M. Okamoto A. Y. Repa J. J. Tu H. Learned R. M. Luk A. (1999). Identification of a nuclear receptor for bile acids. *Science* 284 1362–1365. 10.1126/science.284.5418.1362 10334992

[B99] Marques F. Z. Nelson E. Chu P. Y. Horlock D. Fiedler A. Ziemann M. (2017). High-fiber diet and acetate supplementation change the gut microbiota and prevent the development of hypertension and heart failure in hypertensive mice. *Circulation* 135 964–977. 10.1161/circulationaha.116.024545 27927713

[B100] McIntyre C. W. Harrison L. E. Eldehni M. T. Jefferies H. J. Szeto C. C. John S. G. (2011). Circulating endotoxemia: a novel factor in systemic inflammation and cardiovascular disease in chronic kidney disease. *Clin. J. Am. Soc. Nephrol.* 6 133–141. 10.2215/cjn.04610510 20876680 PMC3022234

[B101] Mencarelli A. Renga B. Distrutti E. Fiorucci S. (2009). Antiatherosclerotic effect of farnesoid X receptor. *Am. J. Physiol. Heart Circ. Physiol.* 296 H272–H281. 10.1152/ajpheart.01075.2008 19028791

[B102] Meyer K. A. Benton T. Z. Bennett B. J. Jacobs D. R. Jr. Lloyd-Jones D. M. (2016). Microbiota-dependent metabolite trimethylamine n-oxide and coronary artery calcium in the coronary artery risk development in young adults study (CARDIA). *J. Am. Heart Assoc.* 5:e003970. 10.1161/jaha.116.003970 27792658 PMC5121500

[B103] Miao J. Ling A. V. Manthena P. V. Gearing M. E. Graham M. J. Crooke R. M. (2015). Flavin-containing monooxygenase 3 as a potential player in diabetes-associated atherosclerosis. *Nat. Commun.* 6:6498. 10.1038/ncomms7498 25849138 PMC4391288

[B104] Midtvedt T. (1974). Microbial bile acid transformation. *Am. J. Clin. Nutr.* 27 1341–1347. 10.1093/ajcn/27.11.1341 4217103

[B105] Miller M. A. McTernan P. G. Harte A. L. Silva N. F. Strazzullo P. Alberti K. G. (2009). Ethnic and sex differences in circulating endotoxin levels: a novel marker of atherosclerotic and cardiovascular risk in a British multi-ethnic population. *Atherosclerosis* 203 494–502. 10.1016/j.atherosclerosis.2008.06.018 18672240

[B106] Mitra S. Drautz-Moses D. I. Alhede M. Maw M. T. Liu Y. Purbojati R. W. (2015). In silico analyses of metagenomes from human atherosclerotic plaque samples. *Microbiome* 3:38. 10.1186/s40168-015-0100-y 26334731 PMC4559171

[B107] Miura K. Ohnishi H. (2014). Role of gut microbiota and Toll-like receptors in nonalcoholic fatty liver disease. *World J. Gastroenterol.* 20 7381–7391. 10.3748/wjg.v20.i23.7381 24966608 PMC4064083

[B108] Miyamoto J. Kasubuchi M. Nakajima A. Irie J. Itoh H. Kimura I. (2016). The role of short-chain fatty acid on blood pressure regulation. *Curr. Opin. Nephrol. Hypertens.* 25 379–383. 10.1097/mnh.0000000000000246 27490782

[B109] Miyazaki-Anzai S. Masuda M. Levi M. Keenan A. L. Miyazaki M. (2014). Dual activation of the bile acid nuclear receptor FXR and G-protein-coupled receptor TGR5 protects mice against atherosclerosis. *PLoS One* 9:e108270. 10.1371/journal.pone.0108270 25237811 PMC4169583

[B110] Mouzaki M. Comelli E. M. Arendt B. M. Bonengel J. Fung S. K. Fischer S. E. (2013). Intestinal microbiota in patients with nonalcoholic fatty liver disease. *Hepatology* 58 120–127. 10.1002/hep.26319 23401313

[B111] Mozaffarian D. Benjamin E. J. Go A. S. Arnett D. K. Blaha M. J. Cushman M. (2016). Heart disease and stroke statistics-2016 Update: a report from the american heart association. *Circulation* 133 e38–e360. 10.1161/cir.0000000000000350 26673558

[B112] Munford R. S. (2016). Endotoxemia-menace, marker, or mistake? *J. Leukoc. Biol.* 100 687–698. 10.1189/jlb.3RU0316-151R 27418356 PMC5014740

[B113] Nagata C. Wada K. Tamura T. Konishi K. Kawachi T. Tsuji M. (2015). Choline and betaine intakes are not associated with cardiovascular disease mortality risk in japanese men and women. *J. Nutr.* 145 1787–1792. 10.3945/jn.114.209296 26063062

[B114] Natarajan N. Hori D. Flavahan S. Steppan J. Flavahan N. A. Berkowitz D. E. (2016). Microbial short chain fatty acid metabolites lower blood pressure via endothelial G protein-coupled receptor 41. *Physiol. Genomics* 48 826–834. 10.1152/physiolgenomics.00089.2016 27664183 PMC6223570

[B115] Neves A. L. Coelho J. Couto L. Leite-Moreira A. (2013). Metabolic endotoxemia: a molecular link between obesity and cardiovascular risk. *J. Mol. Endocrinol.* 51 R51–R64. 10.1530/JME-13-0079 23943858

[B116] Niebauer J. Volk H. D. Kemp M. Dominguez M. Schumann R. R. Rauchhaus M. (1999). Endotoxin and immune activation in chronic heart failure: a prospective cohort study. *Lancet* 353 1838–1842. 10.1016/S0140-6736(98)09286-1 10359409

[B117] Packer C. S. Rice A. E. Johnson T. C. Pelaez N. J. Temm C. J. Potter G. V. (2014). Oxidized low density lipoprotein (OX-LDL) induced arterial muscle contraction signaling mechanisms. *Open Hyperten. J.* 6 20–26. 10.2174/1876526201406010020 23324130

[B118] Parseus A. Sommer N. Sommer F. Caesar R. Molinaro A. Stahlman M. (2017). Microbiota-induced obesity requires farnesoid X receptor. *Gut* 66 429–437. 10.1136/gutjnl-2015-310283 26740296 PMC5534765

[B119] Pedersen H. K. Gudmundsdottir V. Nielsen H. B. Hyotylainen T. Nielsen T. Jensen B. A. (2016). Human gut microbes impact host serum metabolome and insulin sensitivity. *Nature* 535 376–381. 10.1038/nature18646 27409811

[B120] Peluso I. Morabito G. Urban L. Ioannone F. Serafini M. (2012). Oxidative stress in atherosclerosis development: the central role of LDL and oxidative burst. *Endocr. Metab. Immune Disord. Drug Targets* 12 351–360. 10.2174/187153012803832602 23061409

[B121] Philpott D. J. Sorbara M. T. Robertson S. J. Croitoru K. Girardin S. E. (2014). NOD proteins: regulators of inflammation in health and disease. *Nat. Rev. Immunol.* 14 9–23. 10.1038/nri3565 24336102

[B122] Pluznick J. L. (2013). Renal and cardiovascular sensory receptors and blood pressure regulation. *Am. J. Physiol. Renal. Physiol.* 305 F439–F444. 10.1152/ajprenal.00252.2013 23761671 PMC3891255

[B123] Pluznick J. L. Protzko R. J. Gevorgyan H. Peterlin Z. Sipos A. Han J. (2013). Olfactory receptor responding to gut microbiota-derived signals plays a role in renin secretion and blood pressure regulation. *Proc. Natl. Acad. Sci. U.S.A.* 110 4410–4415. 10.1073/pnas.1215927110 23401498 PMC3600440

[B124] Pols T. W. Nomura M. Harach T. Lo Sasso G. Oosterveer M. H. Thomas C. (2011). TGR5 activation inhibits atherosclerosis by reducing macrophage inflammation and lipid loading. *Cell Metab.* 14 747–757. 10.1016/j.cmet.2011.11.006 22152303 PMC3627293

[B125] Qin J. Li R. Raes J. Arumugam M. Burgdorf K. S. Manichanh C. (2010). A human gut microbial gene catalogue established by metagenomic sequencing. *Nature* 464 59–65. 10.1038/nature08821 20203603 PMC3779803

[B126] Rault-Nania M. H. Gueux E. Demougeot C. Demigne C. Rock E. Mazur A. (2006). Inulin attenuates atherosclerosis in apolipoprotein E-deficient mice. *Br. J. Nutr.* 96 840–844. 10.1017/BJN20061913 17092371

[B127] Reijnders D. Goossens G. H. Hermes G. D. Neis E. P. van der Beek C. M. Most J. (2016). Effects of gut microbiota manipulation by antibiotics on host metabolism in obese humans: a randomized double-blind placebo-controlled trial. *Cell Metab.* 24:341. 10.1016/j.cmet.2016.07.008 27508877

[B128] Rey F. E. Faith J. J. Bain J. Muehlbauer M. J. Stevens R. D. Newgard C. B. (2010). Dissecting the in vivo metabolic potential of two human gut acetogens. *J. Biol. Chem.* 285 22082–22090. 10.1074/jbc.M110.117713 20444704 PMC2903421

[B129] Ridlon J. M. Harris S. C. Bhowmik S. Kang D. J. Hylemon P. B. (2016). Consequences of bile salt biotransformations by intestinal bacteria. *Gut Microbes* 7 22–39. 10.1080/19490976.2015.1127483 26939849 PMC4856454

[B130] Russell D. W. (2003). The enzymes, regulation, and genetics of bile acid synthesis. *Annu. Rev. Biochem.* 72 137–174. 10.1146/annurev.biochem.72.121801.161712 12543708

[B131] Sanders M. E. (2008). Probiotics: definition, sources, selection, and uses. *Clin. Infect. Dis.* 46 (Suppl 2), S58–S61; discussion S144–151. 10.1086/523341 18181724

[B132] Santisteban M. M. Qi Y. Zubcevic J. Kim S. Yang T. Shenoy V. (2017). Hypertension-linked pathophysiological alterations in the gut. *Circ. Res.* 120 312–323. 10.1161/circresaha.116.309006 27799253 PMC5250568

[B133] Sayin S. I. Wahlstrom A. Felin J. Jantti S. Marschall H. U. Bamberg K. (2013). Gut microbiota regulates bile acid metabolism by reducing the levels of tauro-beta-muricholic acid, a naturally occurring FXR antagonist. *Cell Metab.* 17 225–235. 10.1016/j.cmet.2013.01.003 23395169

[B134] Senthong V. Li X. S. Hudec T. Coughlin J. Wu Y. Levison B. (2016a). Plasma trimethylamine N-Oxide, a gut microbe–generated phosphatidylcholine metabolite, is associated with atherosclerotic burden. *J. Am. Coll. Cardiol.* 67:2620. 10.1016/j.jacc.2016.03.546 27256833 PMC4893167

[B135] Senthong V. Wang Z. Li X. S. Fan Y. Wu Y. Tang W. H. W. (2016b). Intestinal microbiota-generated metabolite trimethylamine-N-Oxide and 5-year mortality risk in stable coronary artery disease: the contributory role of intestinal microbiota in aCOURAGE-like patient cohort. *J. Am. Heart Assoc.* 5:e002816. 10.1161/JAHA.115.002816 27287696 PMC4937244

[B136] Shih D. M. Wang Z. Lee R. Meng Y. Che N. Charugundla S. (2015). Flavin containing monooxygenase 3 exerts broad effects on glucose and lipid metabolism and atherosclerosis. *J. Lipid Res.* 56 22–37. 10.1194/jlr.M051680 25378658 PMC4274068

[B137] Skoura A. Michaud J. Im D. S. Thangada S. Xiong Y. Smith J. D. (2011). Sphingosine-1-phosphate receptor-2 function in myeloid cells regulates vascular inflammation and atherosclerosis. *Arterioscler. Thromb. Vasc. Biol.* 31 81–85. 10.1161/atvbaha.110.213496 20947824 PMC3013369

[B138] Smits L. P. Kootte R. S. Levin E. Prodan A. Fuentes S. Zoetendal E. G. (2018). Effect of vegan fecal microbiota transplantation on carnitine- and choline-derived trimethylamine-N-oxide production and vascular inflammation in patients with metabolic syndrome. *J. Am. Heart Assoc.* 7:e008342. 10.1161/jaha.117.008342 29581220 PMC5907601

[B139] Staudinger J. L. Goodwin B. Jones S. A. Hawkins-Brown D. MacKenzie K. I. LaTour A. (2001). The nuclear receptor PXR is a lithocholic acid sensor that protects against liver toxicity. *Proc. Natl. Acad. Sci. U.S.A.* 98 3369–3374. 10.1073/pnas.051551698 11248085 PMC30660

[B140] Stoll L. L. Denning G. M. Weintraub N. L. (2004). Potential role of endotoxin as a proinflammatory mediator of atherosclerosis. *Arterioscler. Thromb. Vasc. Biol.* 24 2227–2236. 10.1161/01.ATV.0000147534.69062.dc 15472123

[B141] Studer E. Zhou X. Zhao R. Wang Y. Takabe K. Nagahashi M. (2012). Conjugated bile acids activate the sphingosine-1-phosphate receptor 2 in primary rodent hepatocytes. *Hepatology* 55 267–276. 10.1002/hep.24681 21932398 PMC3245352

[B142] Subah Packer C. (2007). Estrogen protection, oxidized LDL, endothelial dysfunction and vasorelaxation in cardiovascular disease: new insights into a complex issue. *Cardiovasc. Res.* 73 6–7. 10.1016/j.cardiores.2006.11.013 17156767

[B143] Sui Y. Xu J. Rios-Pilier J. Zhou C. (2011). Deficiency of PXR decreases atherosclerosis in apoE-deficient mice. *J. Lipid Res.* 52 1652–1659. 10.1194/jlr.M017376 21685500 PMC3151685

[B144] Szeto F. L. Reardon C. A. Yoon D. Wang Y. Wong K. E. Chen Y. (2012). Vitamin D receptor signaling inhibits atherosclerosis in mice. *Mol. Endocrinol.* 26 1091–1101. 10.1210/me.2011-1329 22638071 PMC3385794

[B145] Tan J. K. McKenzie C. Marino E. Macia L. Mackay C. R. (2017). Metabolite-sensing g protein-coupled receptors-facilitators of diet-related immune regulation. *Annu. Rev. Immunol.* 35 371–402. 10.1146/annurev-immunol-051116-052235 28446062

[B146] Tang W. H. Kitai T. Hazen S. L. (2017). Gut microbiota in cardiovascular health and disease. *Circ. Res.* 120 1183–1196. 10.1161/circresaha.117.309715 28360349 PMC5390330

[B147] Tang W. H. Wang Z. Levison B. S. Koeth R. A. Britt E. B. Fu X. (2013). Intestinal microbial metabolism of phosphatidylcholine and cardiovascular risk. *N. Engl. J. Med.* 368 1575–1584. 10.1056/NEJMoa1109400 23614584 PMC3701945

[B148] Tilg H. Adolph T. E. Gerner R. R. Moschen A. R. (2018). The intestinal microbiota in colorectal cancer. *Cancer Cell* 33 954–964. 10.1016/j.ccell.2018.03.004 29657127

[B149] Townsend M. K. Aschard H. De Vivo I. Michels K. B. Kraft P. (2016). Genomics, telomere length, epigenetics, and metabolomics in the nurses’, health studies. *Am. J. Public Health* 106 1663–1668. 10.2105/ajph.2016.303344 27459442 PMC4981812

[B150] Tremaroli V. Backhed F. (2012). Functional interactions between the gut microbiota and host metabolism. *Nature* 489 242–249. 10.1038/nature11552 22972297

[B151] Troseid M. Ueland T. Hov J. R. Svardal A. Gregersen I. Dahl C. P. (2015). Microbiota-dependent metabolite trimethylamine-N-oxide is associated with disease severity and survival of patients with chronic heart failure. *J. Int. Med.* 277 717–726. 10.1111/joim.12328 25382824

[B152] Vrieze A. Van Nood E. Holleman F. Salojarvi J. Kootte R. S. Bartelsman J. F. (2012). Transfer of intestinal microbiota from lean donors increases insulin sensitivity in individuals with metabolic syndrome. *Gastroenterology* 143 913.e7–916.e7. 10.1053/j.gastro.2012.06.031 22728514

[B153] Wahlstrom A. Sayin S. I. Marschall H. U. Backhed F. (2016). Intestinal crosstalk between bile acids and microbiota and its impact on host metabolism. *Cell Metab.* 24 41–50. 10.1016/j.cmet.2016.05.005 27320064

[B154] Wang H. Zhang W. Zuo L. Dong J. Zhu W. Li Y. (2014). Intestinal dysbacteriosis contributes to decreased intestinal mucosal barrier function and increased bacterial translocation. *Lett. Appl. Microbiol.* 58 384–392. 10.1111/lam.12201 24354719

[B155] Wang Z. Tang W. H. Buffa J. A. Fu X. Britt E. B. Koeth R. A. (2014). Prognostic value of choline and betaine depends on intestinal microbiota-generated metabolite trimethylamine-N-oxide. *Eur. Heart J.* 35 904–910. 10.1093/eurheartj/ehu002 24497336 PMC3977137

[B156] Wang Z. Klipfell E. Bennett B. J. Koeth R. Levison B. S. Dugar B. (2011). Gut flora metabolism of phosphatidylcholine promotes cardiovascular disease. *Nature* 472 57–63. 10.1038/nature09922 21475195 PMC3086762

[B157] Warrier M. Shih D. M. Burrows A. C. Ferguson D. Gromovsky A. D. Brown A. L. (2015). The TMAO-generating enzyme flavin monooxygenase 3 is a central regulator of cholesterol balance. *Cell Rep.* 10.1016/j.celrep.2014.12.036 [Epub ahead of print]. 25600868 PMC4501903

[B158] Watanabe M. Houten S. M. Mataki C. Christoffolete M. A. Kim B. W. Sato H. (2006). Bile acids induce energy expenditure by promoting intracellular thyroid hormone activation. *Nature* 439 484–489. 10.1038/nature04330 16400329

[B159] Watzl B. Girrbach S. Roller M. (2005). Inulin, oligofructose and immunomodulation. *Br. J. Nutr.* 93(Suppl. 1), S49–S55. 10.1079/BJN20041357 15877895

[B160] Wiedermann C. J. Kiechl S. Dunzendorfer S. Schratzberger P. Egger G. Oberhollenzer F. (1999). Association of endotoxemia with carotid atherosclerosis and cardiovascular disease: prospective results from the Bruneck Study. *J. Am. Coll. Cardiol.* 34 1975–1981. 10.1016/S0735-1097(99)00448-9 10588212

[B161] Xu H. Barnes G. T. Yang Q. Tan G. Yang D. Chou C. J. (2003). Chronic inflammation in fat plays a crucial role in the development of obesity-related insulin resistance. *J. Clin. Investig.* 112 1821–1830. 10.1172/JCI200319451 14679177 PMC296998

[B162] Xu X. H. Shah P. K. Faure E. Equils O. Thomas L. Fishbein M. C. (2001). Toll-like receptor-4 is expressed by macrophages in murine and human lipid-rich atherosclerotic plaques and upregulated by oxidized LDL. *Circulation* 104 3103–3108. 10.1161/hc5001.100631 11748108

[B163] Yamashiro K. Tanaka R. Urabe T. Ueno Y. Yamashiro Y. Nomoto K. (2017). Gut dysbiosis is associated with metabolism and systemic inflammation in patients with ischemic stroke. *PLoS One* 12:e0171521. 10.1371/journal.pone.0171521 28166278 PMC5293236

[B164] Yang T. Santisteban M. M. Rodriguez V. Li E. Ahmari N. Carvajal J. M. (2015). Gut dysbiosis is linked to hypertension. *Hypertension* 65 1331–1340. 10.1161/hypertensionaha.115.05315 25870193 PMC4433416

[B165] Yoo J. Y. Kim S. S. (2016). Probiotics and prebiotics: present status and future perspectives on metabolic disorders. *Nutrients* 8:173. 10.3390/nu8030173 26999199 PMC4808900

[B166] Zhang K. Zhang L. Zhou B. Wang Y. Song Y. Rao L. (2012). Lack of association between TLR4 Asp299Gly polymorphism and atherosclerosis: evidence from meta-analysis. *Thromb. Res.* 130 e203–e208. 10.1016/j.thromres.2012.07.008 22857799

[B167] Zhang Y. Wang X. Vales C. Lee F. Y. Lee H. Lusis A. J. (2006). FXR deficiency causes reduced atherosclerosis in Ldlr-/- mice. *Arterioscler. Thromb. Vasc. Biol.* 26 2316–2321. 10.1161/01.atv.0000235697.35431.05 16825595

[B168] Zheng X. Huang F. Zhao A. Lei S. Zhang Y. Xie G. (2017). Bile acid is a significant host factor shaping the gut microbiome of diet-induced obese mice. *BMC Biol.* 15:120. 10.1186/s12915-017-0462-7 29241453 PMC5731064

[B169] Zheng Y. Li Y. Rimm E. B. Hu F. B. Albert C. M. Rexrode K. M. (2016). Dietary phosphatidylcholine and risk of all-cause and cardiovascular-specific mortality among US women and men. *Am. J. Clin. Nutr.* 104:173. 10.3945/ajcn.116.131771 27281307 PMC4919531

[B170] Zhou C. King N. Chen K. Y. Breslow J. L. (2009). Activation of PXR induces hypercholesterolemia in wild-type and accelerates atherosclerosis in apoE deficient mice. *J. Lipid Res.* 50 2004–2013. 10.1194/jlr.M800608-JLR200 19436068 PMC2739759

[B171] Zhu L. Baker S. S. Gill C. Liu W. Alkhouri R. Baker R. D. (2013). Characterization of gut microbiomes in nonalcoholic steatohepatitis (NASH) patients: a connection between endogenous alcohol and NASH. *Hepatology* 57 601–609. 10.1002/hep.26093 23055155

[B172] Zhu L. Zhang D. Zhu H. Zhu J. Weng S. Dong L. (2018). Berberine treatment increases Akkermansia in the gut and improves high-fat diet-induced atherosclerosis in Apoe(-/-) mice. *Atherosclerosis* 268 117–126. 10.1016/j.atherosclerosis.2017.11.023 29202334

[B173] Zhu W. Gregory J. C. Org E. Buffa J. A. Gupta N. Wang Z. (2016). Gut microbial metabolite TMAO enhances platelet hyperreactivity and thrombosis risk. *Cell* 165 111–124. 10.1016/j.cell.2016.02.011 26972052 PMC4862743

[B174] Zhu W. Wang Z. Tang W. H. W. Hazen S. L. (2017). Gut microbe-generated trimethylamine N-oxide from dietary choline is prothrombotic in subjects. *Circulation* 135 1671–1673. 10.1161/circulationaha.116.025338 28438808 PMC5460631

